# Case Report: Mucocele-Like Tumor of the Breast Associated With Ductal Carcinoma *In Situ*

**DOI:** 10.3389/fonc.2022.855028

**Published:** 2022-03-22

**Authors:** Ying Jiang, Li Chai, Dandan Dong, Aamer Rasheed Chughtai, Weifang Kong

**Affiliations:** ^1^ Department of Radiology, Sichuan Academy of Medical Sciences and Sichuan Provincial People’s Hospital, Chengdu, China; ^2^ Department of Pathology, Sichuan Academy of Medical Sciences and Sichuan Provincial People’s Hospital, Chengdu, China; ^3^ Section of Thoracic Imaging, Cleveland Clinic Health System, Cleveland, OH, United States

**Keywords:** mucocele-like tumor, breast, ductal carcinoma *in situ*, case report, imaging

## Abstract

Mucocele-like tumor of the breast is histologically characterized as mucin-containing cysts with mucin leaking to the stroma. It could be associated with atypical ductal hyperplasia (ADH), ductal carcinoma *in situ* (DCIS), and invasive ductal carcinoma (IDC). We report a case of mucocele-like tumor of the breast associated with DCIS confirmed by paraffin section. We review the literature and discuss the imaging features, pathology, and clinical management of the lesion. These lesions demonstrate characteristic imaging features, and we especially highlight the MR characteristics, as they have not been well documented. Performing a diagnostic fine-needle aspiration cytology (FNAC) of mucocele-like tumor carries a risk of tumor underestimation; therefore, excision for all mucocele-like tumors is suggested to be the best approach. However, some recent reports recommend close follow-up for patients with low-risk factors who have mucocele-like tumor without atypia on FNAC.

## Introduction

Mucocele-like tumor of the breast was first described by Rosen in 1986 as mucin containing cysts with extravasated mucin in the stroma (1). Mucocele-like tumor associated with atypical ductal hyperplasia (ADH), ductal carcinoma *in situ* (DCIS), and invasive ductal carcinoma (IDC) were reported subsequently (2–4). In this report, we present imaging features, pathology findings, and clinical management of a 42-year-old woman diagnosed with mucocele-like tumor associated with DCIS. This report aims to illustrate these features and especially highlight the MR characteristics, as these have not been well described previously.

## Case Presentation

A 42-year-old woman accidentally felt a mass in her right breast 2 years ago. The following ultrasound examination reported bilateral cystic dilatation of the ducts (categorized as BI-RADS 2), and routine follow-up was recommended. Two years later, the patient returned to our center right after a heterogeneous irregular mass was revealed on her routine ultrasound follow-up.

**Figure 1:**
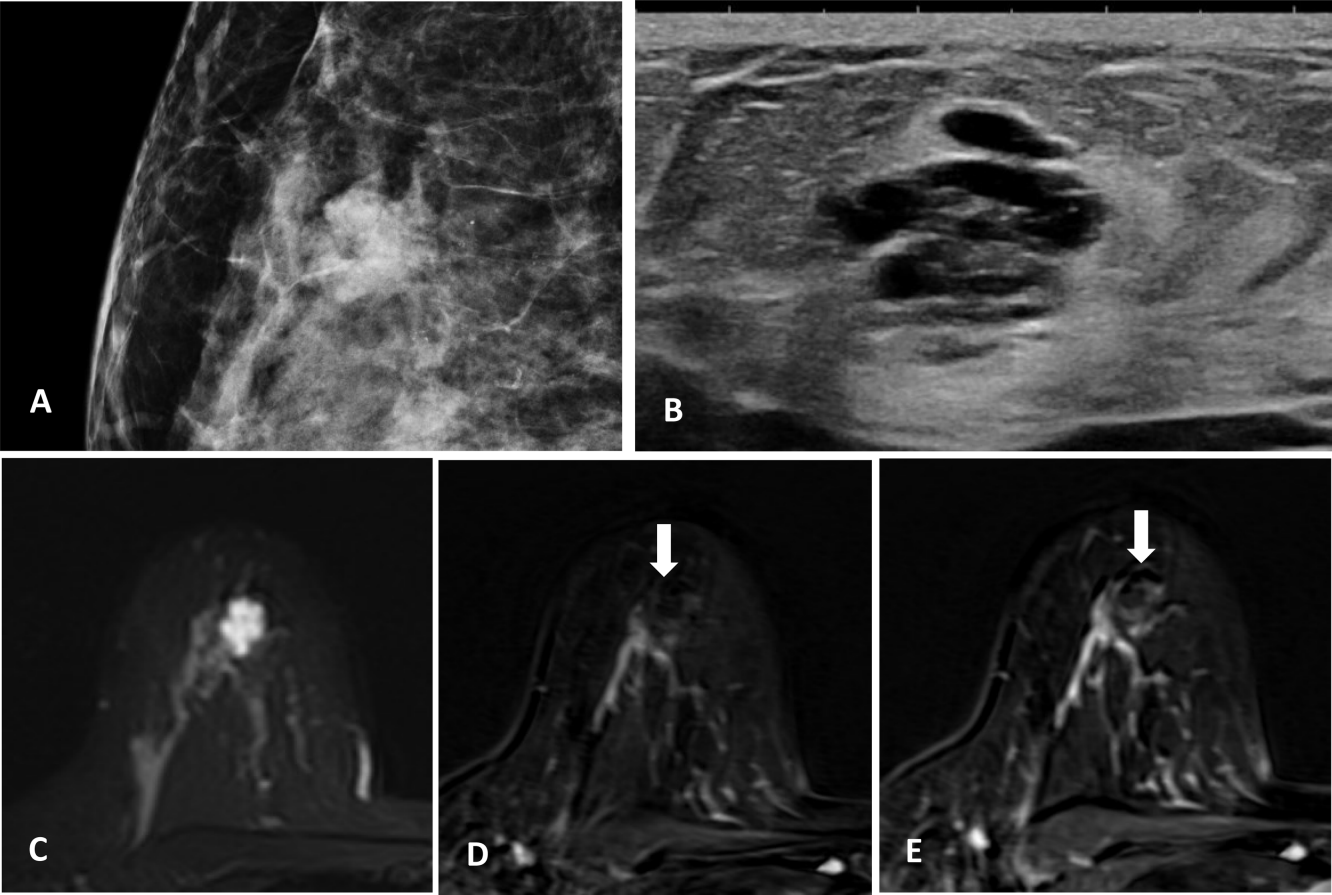
**(A)** Irregular high-density mass with microlobulated margins on mammography, no calcification within the lesion. **(B)** Heterogeneous irregular mass with circumscribed margins on ultrasound. **(C)** MR T2-weighted sequence, the lesion is lobular and shows high signal intensity. **(D, E)** MR-enhanced T1-weighted sequence (immediately after contrast injection and about 6 min delay), the lesion shows persistent slight enhancement at the periphery with no internal enhancement.

Physical examination revealed a 2-cm moderately mobile mass in the right breast with ill-defined margins. Mammography was performed, which demonstrated an irregular high-density mass with microlobulated margin (**Figure 1**). Scattered calcifications were observed in both breasts, but no calcification was found within the lesion. Ultrasound in our center confirmed the presence of a heterogeneous irregular mass with circumscribed margins in the upper outer quadrant of the right breast. Furthermore, the patient underwent an enhanced breast MR examination. The lesion was lobular and showed high signal intensity on T2-weighted sequence, which seemed like clustered cystic lesions. Following Gadolinium enhancement, the lesion showed persistent slight peripheral enhancement but no internal enhancement in all phases.

**Figure 2:**
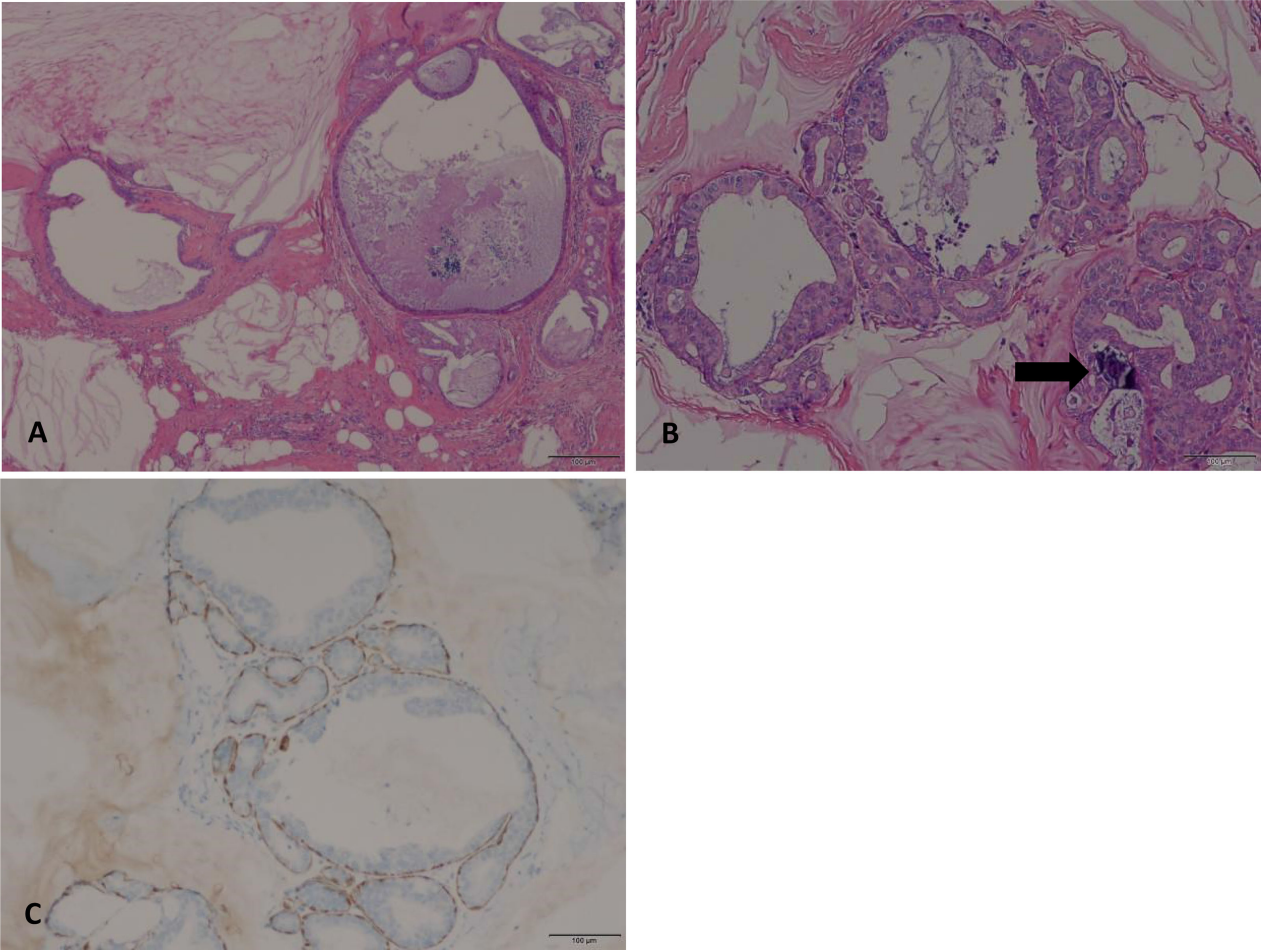
**(A)** Multiple cystic enlarged ducts containing mucinous secretion and extravasated mucin in the stroma (HE, original magnification, 40×). **(B)** Multifocal neoplastic proliferation of epithelial cells (>2mm in size) with low-grade cytological atypia. Microcalcifications are also noted within the enlarged ducts (arrow) (HE, original magnification, 100×). **(C)** Myoepithelial cells at the outer layer of the duct were stained with P63 (original magnification, 100×).

A fine-needle aspiration cytology (FNAC) was obtained, which showed mucocele-like tumor with ADH, and surgical excision was recommended. A partial right mastectomy was then performed. The frozen section showed mucocele-like tumor with atypical intraductal proliferative lesion and microcalcification. A definitive categorization required evaluation of the entire specimen. Paraffin section demonstrated multiple enlarged cystic ducts containing mucinous secretion and extravasated mucin in the stroma (**Figure 2**). Most of the ducts were lined by flat or columnar epithelium cells, and some micropapillary structures were present. In a few areas, we noticed neoplastic proliferations of epithelial cells with cytological atypia. These atypical ductal proliferations were multiple (homogeneous involvement of more than two ducts) and >2 mm in size. The proliferation foci were composed of monomorphic cells with low-grade cytological atypia, locally growing in micropapillae and cribriform. Microcalcifications were also noted within the enlarged ducts. The tumor measured 2 cm in total size (including the mucin-filled ducts without atypical proliferations), and the resection margins were negative. As for immunohistochemical staining, ER (+), PR (+), HER2-negative (focally weak positive), and Ki67 (positive expression rate 1%) immunohistochemistry stains for markers of myoepithelial cells (SMA, CK5/6, Calponin, and P63) confirmed the presence of myoepithelial cells at the outer layer of the ducts. According to the WHO Classification of Tumors of the Breast (1), the diagnosis of mucocele-like tumor associated with low-grade DCIS was established.

In this case, neither radiotherapy nor chemotherapy was indicated because the foci of intraductal carcinoma were not extensive, and the carcinoma did not involve the margins. According to the China Anti-cancer Association breast cancer diagnosis and treatment guideline and criterion (2), mammography and ultrasound were recommended for follow-up. The initial short-term follow-up interval is 3 months in the first 2 years. Assuming stability during this period, the follow-up interval could be increased to 6 months during the third to the fifth year. Again, assuming stability, the follow-up interval could be increased to 1 year for the remainder of her life. An ultrasound follow-up was done 3 months after the surgery, and no tumor recurrence was found.

## Discussion

Mucocele-like tumor is defined as mucin-containing cysts, and the extravasated mucin is commonly present in the stroma. It was first described by Rosen in 1986 as a benign lesion at first (3). In the years that followed, subsequent studies reported mucocele-like tumor associated with ADH, DCIS, and IDC (4–7), indicating that it has a potential for malignancy. Thus, Weaver et al. (8, 9) concluded that mucocele-like tumor and mucinous carcinoma may represent the two ends of pathological spectrum of mucocele-like lesions of the breast.

Recent reports (10, 11) have reached a common consensus that microcalcifications secreted by mucin were the most characteristic finding on mammography. In our case, the lesion appears as a lobulated mass on mammography. We could not observe any calcification within the lesion, even though the photomicrograph suggests that microcalcifications are present. Perhaps, the microcalcifications are too subtle to be detected on routine mammograms. It must be noted here that mammography findings of mucocele-like tumor are nonspecific, especially when there is lack of calcifications (12). It is therefore challenging to diagnose mucocele-like tumor correctly based on mammography features alone. On ultrasound, previous reports suggest that mucocele-like tumors usually manifest as grouped cysts with or without hyperechoic spots (13). The imaging and pathological features in this case demonstrate aggregated clustered cysts, which is consistent with previous studies.

To our knowledge, the MR features of mucocele-like tumor have not been well documented. This case has several MR features. One of the features is the lobular shape, which is consistent with its histological feature of multiple cystic enlarged ducts. Another feature is persistent slight enhancement at the periphery, which could be explained by the gradual movement of contrast medium into the mucin. These features are similar to those of mucinous carcinomas reported previously. Due to the similar histological structure, we hypothesize that the whole spectrum of mucocele-like lesions could have identical characteristics on MRI. This hypothesis still needs to be verified by more cases.

Although there are several MR features of mucocele-like tumor as mentioned above, mucocele-like tumor still needs to be differentiated from other lesions on imaging. The differential diagnosis includes fibroadenoma, pure cyst, and invasive carcinoma of no special type. Sometimes, it would be difficult to identify mucocele-like tumor and fibroadenoma on MRI, especially myxoid fibroadenoma. Both lesions could be lobular, show high signal intensity on T2 weighted sequence, and have persistent enhancement, but fibroadenoma is more likely to be round or oval in shape with a circumscribed margin. Furthermore, in a typical fibroadenoma, calcifications are mainly coarse or “popcorn-like” on mammography, and it has a homogeneous echo-texture on ultrasound (14). A pure cyst is easy to distinguish; it is mostly round or oval in shape with circumscribed margins and does not enhance after contrast injection on MRI. As for invasive carcinoma of no special type, calcifications are usually suspicious on mammography, and the margins are not well-circumscribed. Invasive carcinoma of no special type usually enhances fast on initial phase and has a wash-out kinetic curve (15).

Management of mucocele-like tumor is still not standardized. It is difficult to identify mucocele-like tumor correctly by FNAC because of tumor heterogeneity. Moreover, there is possibility of the presence of ADH, DCIS, or IDC. This case was diagnosed with mucocele-like tumor with ADH by FNAC but upgraded to mucocele-like tumor with DCIS after surgical excision. Previous investigations reported the upgrading rates of mucocele-like tumor on FNAC range from 4% to 30% (16, 17). The potential for upgrading led to recommendations for excision of all mucocele-like tumors. However, recent studies showed that the upgrading rate of mucocele-like tumor without atypia on FNAC is relatively low (<5%). Close clinical and radiological follow-up may be a safe alternative to immediate surgical excision for these patients with low risk factors (18).

## Data Availability Statement

The original contributions presented in the study are included in the article/supplementary material. Further inquiries can be directed to the corresponding author.

## Ethics Statement

Written informed consent was obtained from the individual for the publication of any potentially identifiable images or data included in this article.

## Author Contributions

YJ: writing and editing of the manuscript and review of final submission. LC and DD: collection of data, figure preparation, and review of final submission. AC: editing of the manuscript and review of final submission. WK: collection of data, editing of the manuscript, figure preparation, and review of final submission. All authors contributed to the article and approved the submitted version.

## Conflict of Interest

The authors declare that the research was conducted in the absence of any commercial or financial relationships that could be construed as a potential conflict of interest.

## Publisher’s Note

All claims expressed in this article are solely those of the authors and do not necessarily represent those of their affiliated organizations, or those of the publisher, the editors and the reviewers. Any product that may be evaluated in this article, or claim that may be made by its manufacturer, is not guaranteed or endorsed by the publisher.
